# A Rare Case of Primary Sclerosing Cholangitis Overlapped With Autoimmune Hepatitis and Ulcerative Colitis

**DOI:** 10.7759/cureus.43403

**Published:** 2023-08-13

**Authors:** Saraswathi Lakkasani, Gowthami Sai Kogilathota Jagirdhar, Murad Qirem, Scott W Digiacomo, Srinivasa Pantula

**Affiliations:** 1 Gastroenterology, Saint Michael's Medical Center, Newark, USA; 2 Research, Harvard Medical School, Boston, USA; 3 Internal Medicine, Saint Michael's Medical Center, Newark, USA; 4 Medical Education, Saint Michael's Medical Center, Newark, USA; 5 Gastroenterology and Hepatology, Saint Michael's Medical Center, Newark, USA; 6 General Medicine, Prime Healthcare, Ontario, USA

**Keywords:** autoimmune hepatitis, cholestasis, ursodeoxycholic acid, ulcerative colıtıs, primary sclerosing cholangitis (psc)

## Abstract

Primary sclerosing cholangitis (PSC) is a liver disease of idiopathic origin, displaying a diverse and varied nature, which leads to cholestasis. It is characterized by continuous, advancing inflammation and fibrosis in the bile ducts. PSC is closely linked with inflammatory bowel disease and poses a risk for colon, bile duct, and gallbladder cancer. Unfortunately, there is currently no effective medical treatment available for this condition. In some cases, the disease may progress to end-stage liver failure, making liver transplantation a possible necessity for affected individuals. PSC association with autoimmune hepatitis (AIH) is very rare. This is a case of PSC that is overlapped with AIH. Screening colonoscopy showed colitis, and a biopsy was consistent with ulcerative colitis without any colitis symptoms, emphasizing the need for ruling out any other associated conditions, which respond well to the effective treatment to avoid morbidity and mortality in PSC.

## Introduction

Primary sclerosing cholangitis (PSC) is a chronic liver disease characterized by inflammation and scarring of the biliary ducts, which ultimately leads to strictures. It is often diagnosed in the age range of 30-50, and the association with inflammatory bowel disease (IDB) is a hallmark of the condition, particularly with ulcerative colitis (UC) [1]. The most common presentation of PSC is asymptomatic, but it can present with nonspecific symptoms, such as malaise, pruritus, and right upper abdominal quadrant pain. These symptoms may be masked by the underlying IBD; therefore, a high clinical suspicion for PSC among patients with UC is needed, and vice versa. The association of PSC with autoimmune hepatitis (AIH) is very rare. In this case, we present a 31-year-old female who was diagnosed with PSC overlapped with AIH and UC.

## Case presentation

**Figure 1:**
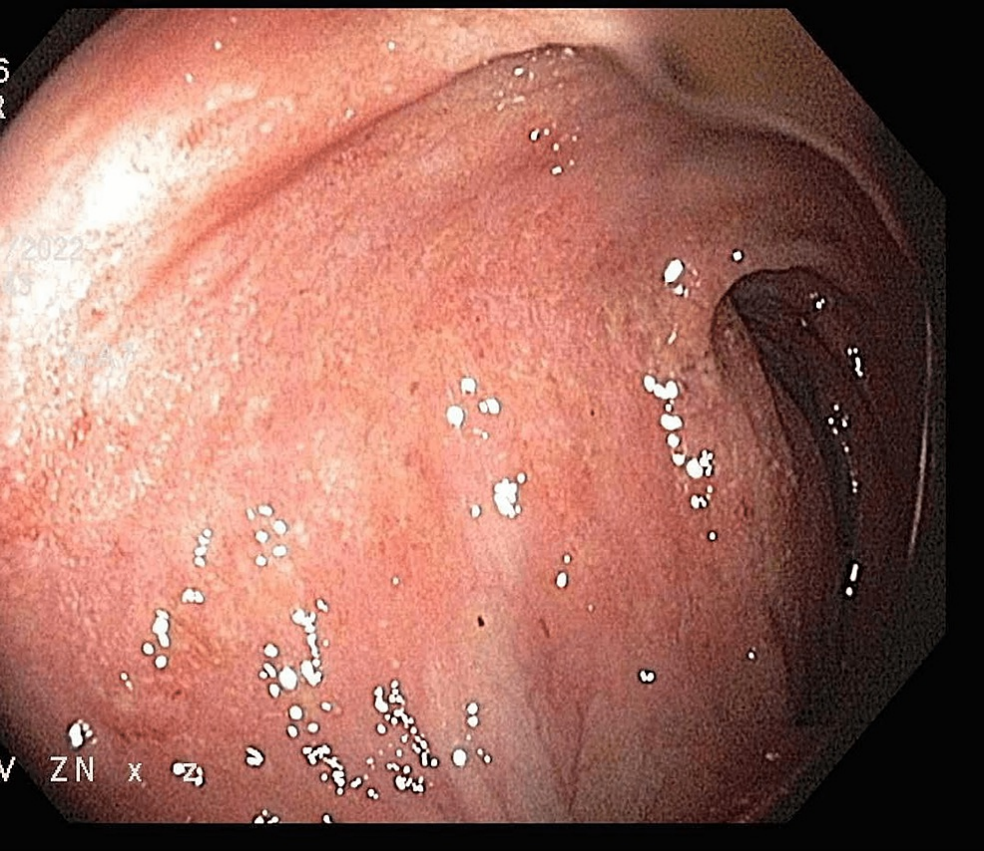
Colonoscopy showing erythema and loss of vascularity in descending colon

**Figure 2:**
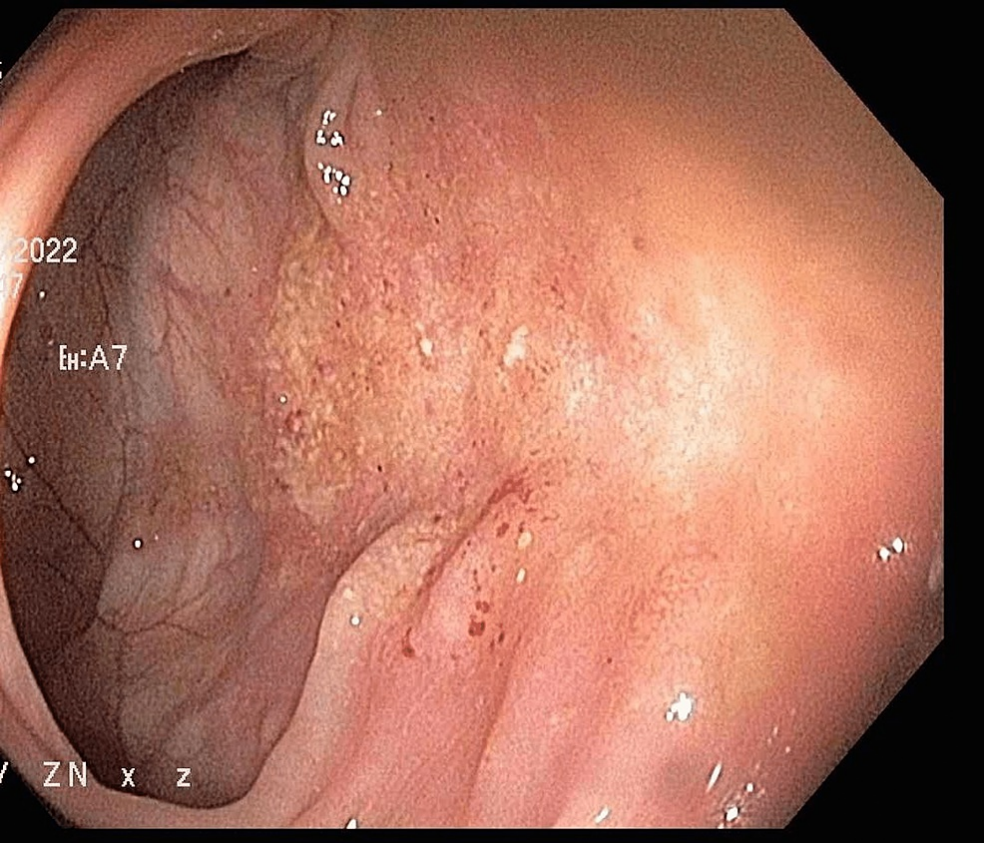
Colonoscopy showing erythema near splenic flexure

**Figure 3:**
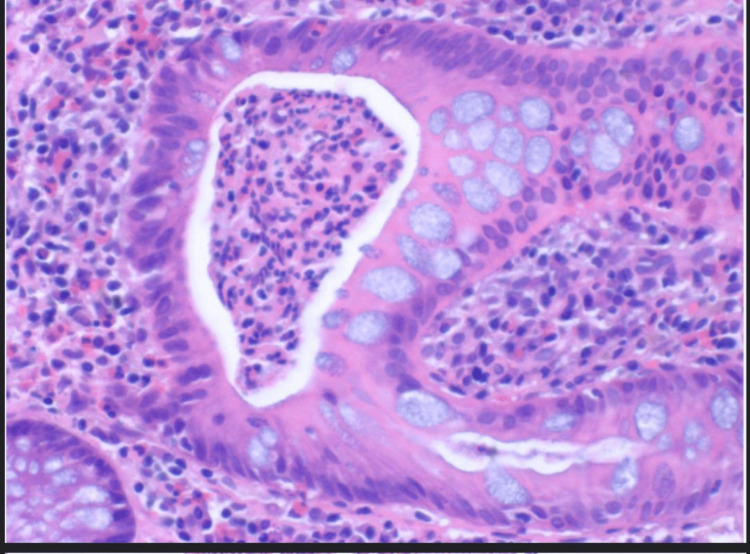
Colonic biopsy showing lymphocytic infiltration in mucosal and submucosa

**Figure 4:**
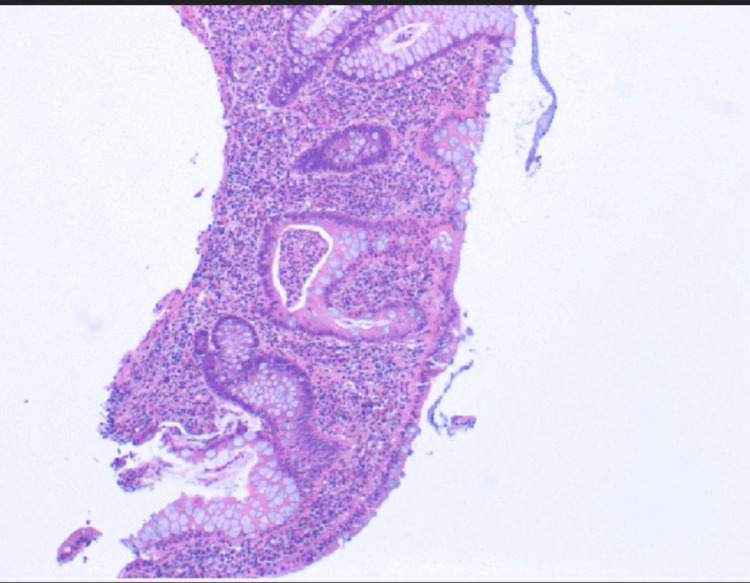
Descending colon biopsy showing inflammatory cells with crypt abscess

A 31-year-old female, with no significant past medical history, was presented to the emergency department with fatigue and 10 Ibs weight loss of three-month duration. Her vital signs were within normal limits. On laboratory workup, she was found to have an elevated alkaline phosphatase of 556 IU/L, aspartate transaminase of 406 IU/L, and alanine transaminase of 279 IU/L. She denied alcohol or drug use. Further workup showed that antinuclear antibodies (ANA), anti-smooth muscle, and antimitochondrial antibodies were negative. An atypical perinuclear anti-neutrophil cytoplasmic antibody (pANCA) titer was 1:640 (indicating the possible presence of associated IBD), and serum immunoglobulin G was elevated at 2,263 mg/dL. We decided to proceed with a colonoscopy, which revealed localized colitis without skip lesions extending from the descending colon to splenic flexure (Figures 1-2). Histopathological examination of the colonic biopsy showed moderate chronic active colitis with crypt abscess but without granulomas. Further immunohistochemistry evaluation of the specimen showed IgG4 scattered cells (Figures 3-4). These findings were consistent with UC.

The laboratory values, combined with the colonoscopy results, made us think of PSC, which is the reason for the abnormal liver function in the setting of newly diagnosed UC. However, MRCP showed no significant abnormalities in the biliary tract. Since there was a high suspicion of PSC despite the negative MRCP, a liver biopsy was done, and chronic portal inflammation with lobular inflammation and periportal fibrosis (stage 2 of 4) and disrupted bile ducts were found, which are findings suggestive of AIH overlapped with PSC. She was treated with a tapering dose of steroids, ursodeoxycholic acid of 300 mg twice daily, and 50 mg of 6-mercaptopurine. Patient symptoms improved, and liver enzymes trended down on follow-up in six weeks.

## Discussion

IBDs, encompassing UC and Crohn’s disease (CD), are frequently associated with various disorders affecting the liver and bile ducts. It is estimated that approximately one-third of individuals with IBD exhibit abnormal biochemical tests, indicating liver involvement in the disease process [2,3]. The underlying causes of hepatobiliary disorders in IBD patients can be categorized into three main groups: manifestations of IBD outside the intestines, medication-related toxicity from treatment, or unrelated primary liver diseases [1,4]. Among these conditions, fatty liver associated with IBD is the most commonly encountered. However, PSC holds the highest level of specificity, as about 70%-80% of patients with PSC also have concurrent IBD. Additionally, around 1.4%-7.5% of individuals with IBD may eventually develop PSC [1,4]. PSC is classified as a rare disease, with an estimated prevalence of 1-16 cases per 100,000 individuals in the United States [5]. Interestingly, PSC can manifest before IBD, which might be diagnosed later, even after a patient undergoes liver transplantation for PSC. On the other hand, PSC can also appear after colectomy in a patient with IBD [6,7]. This interconnection makes it challenging to establish whether PSC patients are free of IBD since the two conditions are commonly associated with each other.

The pathogenesis of PSC is poorly understood, but it is believed to be an immune-mediated process, as it is related to IBD, which is an autoimmune disorder [8]. Laboratory tests usually show elevated determinations of alkaline phosphatase, gamma-glutamyltransferase, and total bilirubin. PSC progression is slow and highly variable, and it can eventually lead to cirrhosis, leading to portal hypertension and its associated complications, with a 10-year survival of approximately 65% [9,10]. Problems related to PSC can also develop, such as recurrent ascending cholangitis and cholangiocarcinoma; in a multi-center study of 7,119 PSC patients, hepatobiliary malignancy was diagnosed in 10.9% [11]. In addition, the risk of colon cancer increases in patients diagnosed with PSC. It was found that colorectal cancer is fivefold higher than in IBD without PSC. Hence, colonoscopic surveillance should be performed regularly from the time of diagnosis of PSC [12,13].

Our case is a rare entity with PSC combined with AIH in association with UC. The patient is treated with steroids for AIH, and ursodeoxycholic acid is added given a cholestatic pattern of the liver enzymes, which trended down eventually, and UC was treated with 6-mercaptopurine.

## Conclusions

High suspicion of PSC should go for a liver biopsy even though MRCP is negative as early diseases may not show changes in MRCP and workup-associated disorders are needed as a part of management in PSC even though the patient has no associated symptoms. Our case emphasizes the importance of a liver biopsy and the need for colonoscopy in PSC patients even if the patient has no symptoms. Likewise, when evaluating UC patients with cholestasis, it is essential to consider PSC as a potential differential diagnosis since PSC can be asymptomatic. It is recommended to include magnetic resonance cholangiopancreatography (MRCP) as part of the diagnostic workup since it can aid in the early detection of PSC. However, it can sometimes be negative, and if we have a high suspicion of PSC, a further diagnostic workup is needed. It is also essential to do colonoscopy in all PSC patients even in asymptomatic patients because of its potential association with IBD.
